# The Antioxidant Effect of Erythropoietin on Thalassemic Blood Cells

**DOI:** 10.1155/2010/978710

**Published:** 2010-12-21

**Authors:** Johnny Amer, Mutaz Dana, Eitan Fibach

**Affiliations:** Department of Hematology, Hadassah-Hebrew University Medical Center, Ein-Kerem, 91120 Jerusalem, Israel

## Abstract

Because of its stimulating effect on RBC production, erythropoietin (Epo) is used to treat anemia, for example, in patients on dialysis or on chemotherapy. In *β*-thalassemia, where Epo levels are low relative to the degree of anemia, Epo treatment improves the anemia state. Since RBC and platelets of these patients are under oxidative stress, which may be involved in anemia and thromboembolic complications, we investigated Epo as an antioxidant. Using flow-cytometry technology, we found that in vitro treatment with Epo of blood cells from these patients increased their glutathione content and reduced their reactive oxygen species, membrane lipid peroxides, and external phosphatidylserine. This resulted in reduced susceptibility of RBC to undergo hemolysis and phagocytosis. Injection of Epo into heterozygous (Hbb*^th3/+^*) *β*-thalassemic mice reduced the oxidative markers within 3 hours. Our results suggest that, in addition to stimulating RBC and fetal hemoglobin production, Epo might alleviate symptoms of hemolytic anemias as an antioxidant.

## 1. Introduction

Erythropoietin (Epo), a hormone released upon hypoxia mainly in the kidneys, enhances red blood cell (RBC) production (erythropoiesis) by stimulating the proliferation of erythroid progenitors and precursors in the bone marrow [1, 2]. This effect is mediated by a homodimeric Epo receptor (EpoR), a class 1 cytokine receptor [3]. Recombinant human Epo is widely used for the treatment of anemia, for example, in patients on chemotherapy [4] or on hemodialysis [5–7]. Treatment with Epo was also tried experimentally in patients with thalassemia [8–11]. In these patients, in spite of the state of chronic anemia, the level of Epo is usually low relative to the degree of anemia [12, 13]. Administration of Epo to splenectomized patients with *β*-thalassemia intermedia resulted in a dose-dependent improvement in their anemia [14, 15]. A long-acting Epo (darbepoetin alfa) was shown to substantially increase hemoglobin (Hb) levels in patients with HbE-*β*-thalassemia [16].

Although the main effect of Epo is related to stimulation of erythropoiesis, it was suggested that in patients with chronic renal failure on dialysis its anti anemia effect may also be associated with increasing the survival of mature red blood cells (RBC) [17]. It was reported that in such patients, the increased number of RBC exhibiting surface phosphatidylserine (PS), a marker of senescence [18–20], was reduced within 4 hrs after administration Epo [20]. PS is increased in RBC following several stress situations, including oxidative stress [21]. We have shown that RBC and platelets from patients with *β*-thalassemia are under oxidative stress; they demonstrate an increased reactive oxygen species (ROS) and a decreased content of reduced glutathione (GSH), the main cellular antioxidant, than their normal counterparts [22]. This oxidative stress resulted in membrane lipid peroxidation and exposure of PS. The latter is considered a major factor in shortening the life-span of RBC [18] and hyperactivation of platelets from thalassemic patients [23].

We now investigated the in vitro effect of Epo on the oxidative status of RBC and platelets from *β*-thalassemic patients and the in vivo effect on these cells of Epo administration in *β*-thalassemic mice. The results show that Epo has an antioxidative effect in both RBC and platelets by which it may benefit thalassemic patients.

## 2. Materials and Methods

### 2.1. Patients' Blood Samples

Peripheral blood samples were obtained from normal donors and patients with *β*-thalassemia intermedia and major. The patients' mutations and some relevant clinical parameters (e.g., transfusion and chelation therapy) were recently summarized [24]. In polytransfused patients, blood samples were obtained before transfusion, that is, at least 3 weeks following the previous transfusion. These experiments were approved by the Hadassah-Hebrew University Medical Center Ethical Committee of Human Experimentation and are in accordance with the Helsinki Declaration of 1975. Informed consent was obtained in all cases.

Blood samples were collected in EDTA-containing tubes (Becton Dickinson, Plymouth, UK) and were washed twice and resuspended in Ca^++^- and Mg^++^-free Dulbecco's Phosphate Buffered Saline (PBS) (Biological Industries, Kibbutz Beit-Haemek, Israel) to a concentration of 4 × 10^6^ RBC/ml. Within 2 hrs of blood withdrawal, the cells were incubated at 37°C in a 5% CO_2_ incubator with human recombinant erythropoietin (Epo, Cilag AG, Schaffhausen, Switzerland) at doses and duration indicated in the text.

### 2.2. Mice

The founders of a thalassemic mouse colony were obtained from Dr. S. Rivella, Weill Medical College of, Cornell University, NY, NY. Heterozygous (Hbb*^th3/+^*) mice, exhibit severe anemia (7 to 9 g/dL Hb), abnormal RBC morphology, splenomegaly, and hepatic iron deposition [25]. Animals were bred at the animal facility of the Sharett Institute, Hadassah Hospital, Jerusalem, Israel. Four-month-old mice were intraperitoneally inoculated with Epo (5 000 U/kg). Blood samples (20 *μ*L) were collected from their tail vein before and 2 hrs after treatment. These experiments were approved by the Hadassah—Hebrew University Medical Center Animal Ethics Committee.

### 2.3. Assays for RBC Hemolysis and Phagocytosis

RBC (5 × 10^6^/mL) were washed and suspended in PBS, and incubated overnight. RBC were then centrifuged, resuspended in PBS and counted. Hemolysis was calculated as percentage of lysed RBC compared with the RBC input. The results were confirmed by spectrophotometric measurement [26] of the Hb content in the hemolysate.

To measure phagocytosis, 5 × 10^6^/mL RBC diluted in PBS were added to macrophage cultures prepared as previously described [24]. After overnight incubation at 37°C, the nonphagocytosed RBC were harvested by careful washing and counted microscopically using a hemocytometer. The percent of phagocytosed RBC was calculated per the RBC input.

### 2.4. Flow Cytometry Measurements of Oxidative Stress Markers

Oxidative stress markers were measured as previously described [22, 27, 28] in mixtures of RBC and platelets. ROS was measured following incubation for 15 min with 0.4 mM 2′-7′-dichlorofluorescin diacetate (DCF) (Sigma, St. Louis, MO). Membrane lipid peroxides were measured following 1-hour incubation with 40 *μ*M N-(fluorescein-5-thiocarbamoyl)1,2-dihexadecanoyl-sn-glycero-3-phosphoethanolamine, triethylammonium salt (fluor-DHPE) (Molecular Probes Inc., Eugene, OR). For measurement of calcein uptake, the cells were incubated for 15 min with 0.5 *μ*M calcein acetoxymethyl ester (Sigma). Incubations were carried out at 37°C in a humidified 5% CO_2_ incubator. For measuring external phosphatidylserine (PS) the cells were washed and resuspended in 100 *μ*L calcium-binding buffer ((10 mM HEPES, 140 mM NaCl and 2.5 mM CaCl_2_ (pH 7.4)) and stained for 20 min at room temperature with 5 *μ*L FITC-Annexin V (IQ products, Groningen, The Netherlands).

The GSH content was measured by spinning the cells down and incubating the pellet for 3 min. at room temperature with 40 *μ*M (final concentration) of mercury orange (Sigma). A 1 mM stock solution of mercury orange was made up in acetone and stored at −20°C. The cells were then washed and resuspended in PBS.

Following treatments as indicated above the cells were analyzed with a Fluorescence-Activated Cell Sorter (FACS-calibur, Becton-Dickinson, Immunofluorometry systems, Mountain View, CA). Instrument calibration and settings were performed using CaliBRITE-3 beads (Becton-Dickinson). The cells were passed at a rate of ~1000 per second, using saline as the sheath fluid. A 488 nm argon laser beam was used for excitation. RBC and platelets were gated based on their size (forward light scatter, FSC) and granularity (side light scatter, SSC) as previously described [27]. The identity of each cell population was verified by staining with antibodies to glycophorin-A and CD41 for RBC and platelets, respectively [29]. For every assay, unstained cells, both treated and nontreated, were used as controls. The Mean Fluorescence Intensities (MFIs) and the percentages of positive cells were calculated using the FACS-equipped CellQuest^R^ software. The results are expressed as the average ± standard deviation (SD) MFI and compared using the two-sample Student's *t*-test for differences in means.

## 3. Results

**Figure 1:**
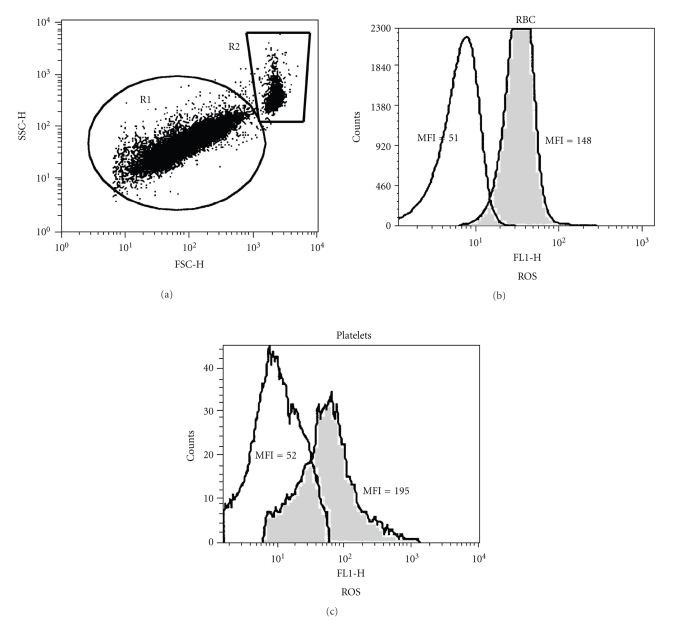
Flow cytometry analysis of the Epo effect on ROS generation by RBC and platelets. A diluted blood sample obtained from a thalassemia patient was treated for 2 hrs with Epo (1 U/ml) at 37°C, stained with DCF and then stimulated with 1 mM H_2_O_2_ for 15 min. (a) FCS versus SSC dot plot. The gates indicate the position of platelets (R1) and RBC (R2). (b-c) Distribution histograms showing DCF-derived fluorescence (FL-1) of untreated (grey) and Epo-treated (white) RBC (b) and platelets (c). The mean fluorescent intensity (MFI) of each population is indicated.

The flow cytometry analysis of the influence of Epo on the intracellular content of ROS in thalassemic RBC and platelets is exemplified in Figure 1. Diluted blood samples were treated with Epo (1 U/ml) for 2 hrs at 37°C, stained with DCF and then stimulated with H_2_O_2_ (1 mM).  Figure 1(a) shows a FSC × SSC dot plot. Gates were set on platelets (R1) and RBC (R2) based on their size (FSC) and granularity (SSC). The DCF-fluorescence histograms of the gated RBC (Figure 1(b)) and platelets (Figure 1(c)), untreated (grey histograms) or treated with Epo (white histograms), as well as their MFI are shown. Epo-treated RBC and platelets in this sample had 2.9-fold and 3.75-fold lower ROS levels, respectively, compared with nontreated cells.

**Figure 2:**
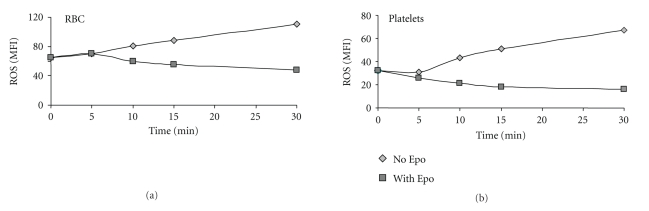
The kinetics of EPO effect on ROS generation by RBC and platelets. A diluted blood sample obtained from a thalassemia patient was stained with DCF, washed, and then incubated at room temperature with Epo (1 U/ml). Fluorescence was measured by flow cytometry at the indicated time points. The time-related changes in the mean fluorescent intensity (MFI) of each population are indicated.

A representative kinetics experiment of Epo effect on ROS generation by RBC and platelets is presented in Figure 2. A blood sample obtained from a thalassemia patient was stained with DCF, washed, and then incubated at room temperature with Epo (1 U/ml). The time-related changes in the fluorescence of each population are indicated. The results indicate that the antioxidative effect of Epo starts within 10–15 min. Similar results were obtained in 3 additional experiments with cells derived from different patients. 

**Figure 3:**
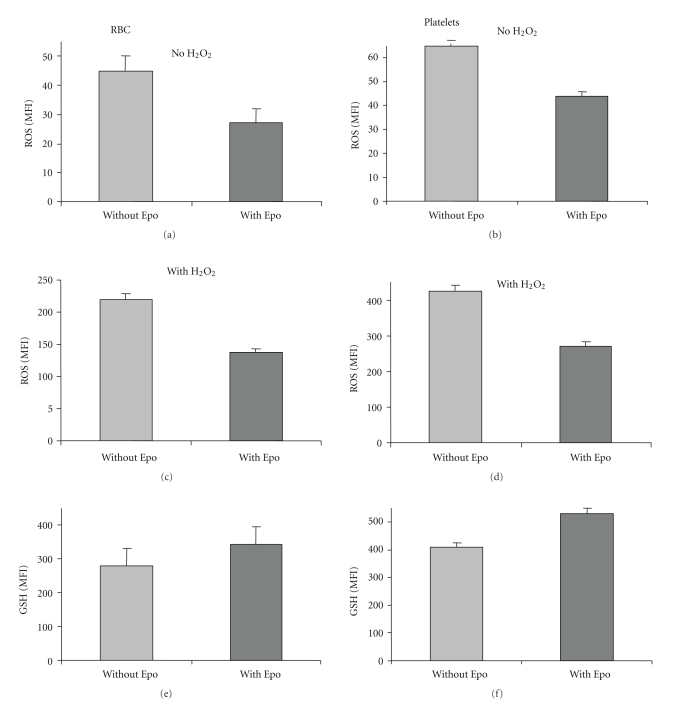
Effect of Epo on the oxidative stress of RBC and platelets from thalassemic patients. Diluted blood samples were untreated with or without 1 U/ml Epo for 3 hrs at 37°C, then stimulated (a, b) or not (c, d) with 1 mM H_2_O_2_ for 15 min, and assayed for ROS. RBC (e) and platelets (f) treated with or without Epo were also assayed for GSH. The results, presented as the average (*N* = 11) of the mean fluorescence index (MFI) ± SD, show a decrease in ROS (*P* < .05) and an increase in GSH (*P* < .05) following Epo treatment.

**Figure 4:**
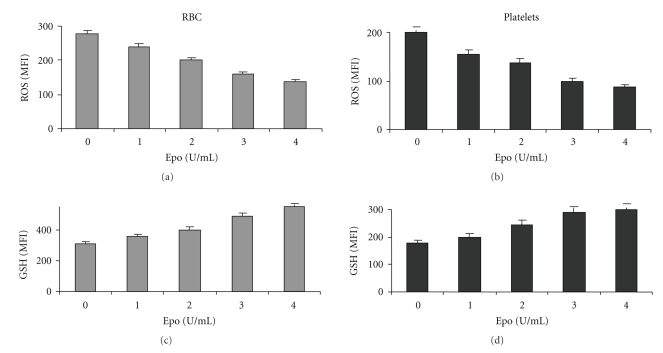
The Epo dose-effect relationship on ROS and GSH of thalassemic RBC and platelets. Diluted blood samples were exposed to different concentrations of Epo for 2 hrs at 37°C and then stimulated with 1 mM H_2_O_2_ for 15 min. The results show the average (*N* = 4) mean fluorescence index (MFI) ± SD of ROS and GSH in RBC and platelets.

The effect of Epo on ROS and GSH of blood cells obtained from 11 patients with *β*-thalassemia is summarized in Figure 3. On the average, Epo reduced ROS in RBC and platelets by 1.5- to 2-fold (*P* < .005) (Figures 3(a) and 3(b)). The effect was noted in nonstimulated (Figures 3(a) and 3(b)) and H_2_O_2_-stimulated cells (Figures 3(c) and 3(d)), indicating that Epo decreased the cells' basal ROS as well as their ability to generate ROS in response to an oxidant. The figure also shows that Epo treatment increased the GSH levels by 1.25-fold in both RBC and platelets (*P* < .01) (Figures 3(e) and 3(f)).  Figure 4 shows that the effects of Epo on thalassemic RBC and platelets are dose-dependent.

**Figure 5:**
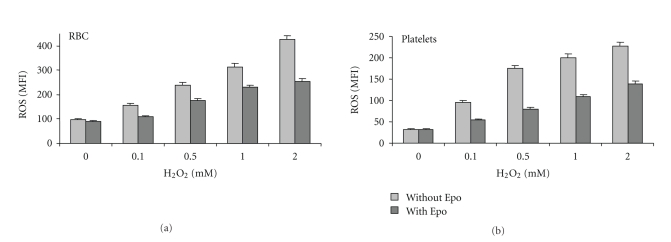
Effect of Epo on ROS production by H_2_O_2_-stimulated normal RBC and platelets. Diluted blood samples were treated with the indicated concentrations of H_2_O_2_ for 30 min, then incubated with or without Epo (2 U/ml) for additional 2 hrs, washed and assayed for ROS. The results show the average (*N* = 3) mean fluorescence index (MFI) ± SD.

Oxidative stress can be induced in normal RBC and platelets by treatment with oxidants [22, 27]. To study the effect of Epo on such cells, normal blood samples were treated for 30 min with different concentrations of H_2_O_2_ and then were treated or not with Epo (2 U/ml) for an additional 2 hrs.  Figure 5 shows that H_2_O_2 _dose-dependently increased ROS and that Epo significantly inhibited this effect of H_2_O_2 _in both normal RBC and platelets.

**Figure 6:**
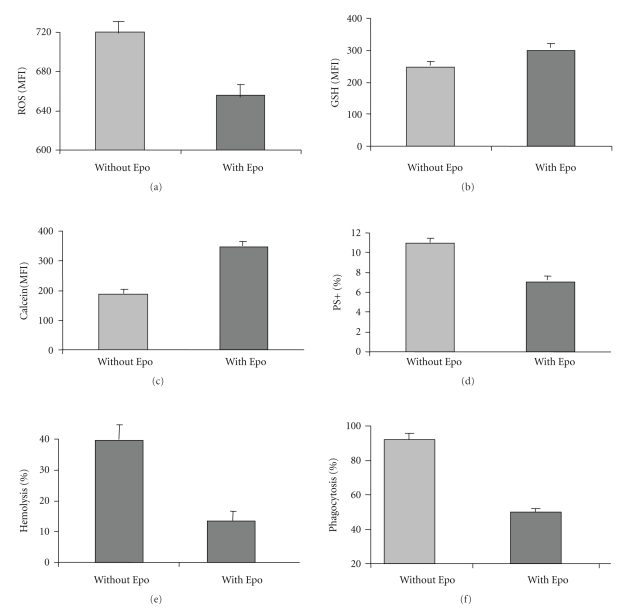
The long-term effects of Epo on RBC oxidative markers, ageing, and susceptibility to hemolysis, and phagocytosis. Thalassemic RBC were diluted to 4 × 10^6^/ml with their plasma and incubated with or without Epo (2 U/ml) for 3 days. The cells were then harvested and assayed for ROS (a), GSH (b), calcein (c), external phosphatidylserine (PS) (d), hemolysis (e) and phagocytosis (f) as detailed in Section 2. The results in (a), (b) and (c) are presented the mean fluorescence index (MFI), in (d) as the percentage of PS positive RBC, and in (e) and (f) as the percentage of hemolysed and phagocytosed RBC, respectively, compared to the RBC input. The data are the average ± SD of 4 experiments performed with blood samples derived from different patients.

In vivo, oxidative stress in RBC is associated with accelerated senescence, increased intrasvascular hemolysis, and mainly extravascular hemolysis [30]. We correlated the effects of Epo on ROS and GSH of RBC with its effects on calcein staining and PS exposure, markers of senescence [18, 31], and susceptibility to undergo hemolysis and phagocytosis. Thalassemic RBC were diluted and incubated in their autologous plasma for 3 days with or without Epo (2 U/ml). The results (Figure 6) show that concomitant with the decrease in ROS (Figure 6(a)) and the increase in GSH (Figure 6(b)), Epo treatment increased their staining with calcein (by 190%) (Figure 6(c)) and decreased their exposure of PS (Figure 6(d)) (by 40%), hemolysis (70%) (Figure 6(e)) and phagocytosis (80%) (Figure 6(f)). These results were highly significant (*P* < .005).

**Figure 7:**
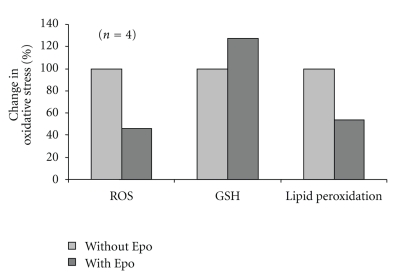
Effect of Epo on thalassemic mice. Heterozygous *β*-thalassemic intermedia (Hbb*^th3/+^*) mice (*N* = 4) with low hemoglobin levels (7–9 g/dL) were inoculated (i.p) with Epo (5000 U/kg). Before (Without Epo) and 2 hrs after (With Epo) injection, blood was drawn and RBC were assayed. The changes in the indicated parameters are shown. Values of untreated mice (Without Epo) were taken as 100%.

The in vivo effect of Epo was determined in thalassemic mice. Blood samples were drawn prior and 2 hours after i.p. injection of Epo (5000 U/kg), and RBC were analyzed for ROS, GSHs and lipid peroxidation. The results demonstrated that Epo treatment significantly reduced ROS and lipid peroxidation and enhanced the GSH level (Figure 7), indicating its ability to ameliorate oxidative stress parameters in vivo.

## 4. Discussion

In the *β*-hemoglobinopathies, *β*-thalassemia, and sickle cell disease, although the primary lesion is in the *β*-globin gene, the damage to the RBC is mediated in part by oxidative stress [32, 33]. It has been previously shown that in these diseases RBC are at oxidative stress as a result of their unstable Hb and iron overload, caused by increased absorption and blood transfusions [34, 35]. Using flow cytometry, we demonstrated higher ROS generation and lower GSH content in these cells compared with normal RBC at basal level, as well as following oxidative insult, such as treatment with H_2_O_2_ [22, 36]. These effects were associated with RBC membrane changes, including lipid peroxidation and externalization of PS [37, 38] and resulted in increased susceptibility to hemolysis and to phagocytosis by macrophages [24], resulting in short survival of the RBC in the circulation and subsequently in chronic anemia. Oxidative stress was also found in the platelets of these patients [22]. Since oxidative stress has been associated with platelets' activation [39], this may explain, in part, the tendency of these patients to develop thromboembolic complications [23].

We now report that Epo can ameliorate the oxidative stress and some of its consequences in RBC and platelets in thalassemia. Epo is being used to treat chronic anemia in a variety of clinical conditions, such as the myelodysplastic syndrome [40], oncology patients undergoing chemotherapy [4] and patients with chronic renal failure undergoing hemodialysis [5–7]. Although the main effect of this treatment is to increase the RBC mass by stimulating erythropoiesis, some studies suggest that Epo may also directly affect mature RBC: Myssina et al. [20] have shown that Epo inhibits RBC Ca^2+^ channels with subsequent reduction in PS exposure, and that intravenous administration of Epo to dialysis patients decreased within 4 hrs the frequency of RBC with exposed PS. Clinical data in such patients further revealed that Epo acts as a survival factor for mature RBC by extending their life [17].

In *β*-thalassemia, Epo treatment was shown to improve the state of anemia [8, 11, 14]. The rational of this treatment in thalassemia is twofold: to stimulate erythropoiesis and to elevate the production of fetal Hb; the latter compensates for the lack or reduced content of HbA [41]. However, in these patients Epo stimulates thalassemic erythropoiesis with production of abnormal RBC having excess *α*-globin chains, membrane damage, and short survival. As for stimulation of fetal Hb, contradictory results were reported [8, 42, 43]. Our findings raise the possibility that Epo administration may benefit thalassemic patients also by reducing oxidative stress and thereby prolonging the survival of their RBC as well as lowering the state of activation of their platelets.

Epo is known to have a protective effect in nonerythroid cells, such as neuronal cells and cardiomyocytes [44]. For example, significant improvement was demonstrated in stroke patients who were administered Epo within 8 hrs of the onset of symptoms [45]. The mechanism of Epo-induced protection in nonerythroid cells was reported to involve a number of signaling pathways, including the Jak-2/STAT [46], a crucial pathway of its erythropoietic effect [47]. However, the effect of Epo in non-erythroid cells is probably unrelated to its influence in erythropoiesis. The effect on erythropoiesis requires the continuous presence of Epo, whereas a brief exposure is sufficient for neuroprotection [48]. Consequently, desialylated Epo, which has the same affinity to the Epo receptor (EpoR) but a reduced erythropoietic effect due to its short life-span, remains neuroprotective [49]. Carbamylated Epo (CEpo), another Epo analog, which does not bind to EpoR and lacks erythropoietic activity, confers neuroprotection and cardioprotection against various cellular injuries [50–52]. Our preliminary results suggest that for the antioxidative effect in thalassemic RBC continuous Epo exposure is not required and that CEpo is active (data not shown).

The receptor complex mediating the Epo protective effects in non-erythroid cells differs from EpoR with respect to the affinity for Epo, molecular weight, and associated proteins (reviewed in [53]). It was suggested that the protective effect of CEpo is mediated through a hetero-receptor complex comprising of EpoR and a *β*-receptor subunit (CD131), a signal-transducing subunit shared by receptors to several cytokines [53].

Several reports attributed the protection by Epo of non-erythroid cells to its anti-oxidative effect, for example, [54–58]. In addition, Epo has been also shown to affect oxidative parameters of mature RBC. Thus, starvation, which was found to deplete the endogenous Epo, increased lipid peroxidation of the RBC membrane, whereas administration of Epo reversed the effect [59]. Epo treatment of hemodialysis patients resulted in reduced lipid peroxidation and enhanced SOD, catalase, and other antioxidant activities [60–65]. These effects of Epo could be related to its influence during RBC production (erythropoiesis). Moreover, improved antioxidant status following Epo treatment of newborn rabbits was suggested to be caused indirectly by utilization of the oxidative active serum iron by developing erythroid precursors, thus making it unavailable for generation of oxygen radicals via the Fenton reaction [66]. In the present study we report a direct effect of Epo on RBC, which is unrelated to erythropoiesis. The effects were observed in vitro by incubating peripheral blood RBC with Epo as well as shortly (3 hrs) after injection of Epo into *β*-thalassemic mice. These results are in agreement with reports by Datta et al. who showed that Epo has multiple effects on mature RBC, including an anti-oxidative one [37, 67–72].

The mechanism underlying the short-term effect of Epo on the oxidative stress of mature RBC is not clear. Epo affects erythroid cells through their surface Epo-R, but mature RBC (and reticulocytes) appear to lack Epo-R [73, 74]. However, it was reported that Scatchard plot analysis of radio-labeled-Epo binding disclosed a low, albeit detectable, number of Epo-binding sites on RBC with high affinity to Epo [20] that was similar to that of the Epo-R of early erythroid progenitors [73]. Interaction of Epo with the mature RBC membrane was also reported by Baciu et al. [75]. To probe the possibility that Epo-R is involved in mediating the effect of Epo on RBC, we inhibited Jak-2, a crucial step in the signal transduction pathway of Epo-R, by Jak inhibitor I [76]. Treatment of thalassemic RBC with this inhibitor did not inhibit the antioxidative effect of Epo (data not shown).

The protective effect of Epo may be mediated through scavenging activity in the extracellular milieu: Epo contains more basic than acidic amino acids and many charged residues [77] that may act as a “sink” for ROS (e.g., hydroxyl radicals) [78]. Moreover, Epo is a highly sialidated glycoprotein [79]. It has been recently reported that mucin, a typical sialic acid containing high-molecular weight glycoprotein, is an anti-oxidant and that sialic acid is crucial for this activity [80]. Sialic acid may also function intracellularly. Oetke et al. [81], using human hematopoietic cell lines which are hyposialylated due to a deficiency in *de novo* sialic acid biosynthesis, demonstrated efficient uptake and incorporation of free sialic acid. Other studies reported that human thalassemic RBC have a lower content of sialic acid than normal RBC [82, 83], and that sialic acid can be taken up by human RBC [84, 85]. In our experiments, using several methodologies, Epo, at the concentrations tested in this study, did not demonstrate any ROS scavenging activity in a cell-free system (data not shown).

In the present study we report the antioxidative potential of Epo on RBC and platelets. Indeed, its activity was demonstrated at concentrations far above normal serum levels, but this does not rule out a role at physiological concentrations—continuous, accumulative subthreshold effects, which could not be detected by the methodology used, may be of physiological importance. Epo is obviously inappropriate as an anti-oxidative drug: it is less potent and much more expensive than other anti-oxidants and, in addition, has to be administrated by injection. Nevertheless, under conditions when its levels are very high, such as during severe anemia, for example, following massive bleeding or in aplastic anemia, or following its administration to patients on hemodialysis or chemotherapy, its potential protective effect as an antioxidant on RBC and platelet survival should be considered.
